# Supplementary material for: Peptide Nucleic Acid (PNA)-Enhanced Specificity of a Dual-Target Real-Time Quantitative Polymerase Chain Reaction (RT-qPCR) Assay for the Detection and Differentiation of SARS-CoV-2 from Related Viruses
Source: Diagnostics (Basel). 2020 Sep 30;10(10):775. doi: 10.3390/diagnostics10100775 (PMC7601008; doi:10.3390/diagnostics10100775)
Supplement: Supplementary file 1 [file diagnostics-10-00775-s001.pdf]

Supplementary Materials

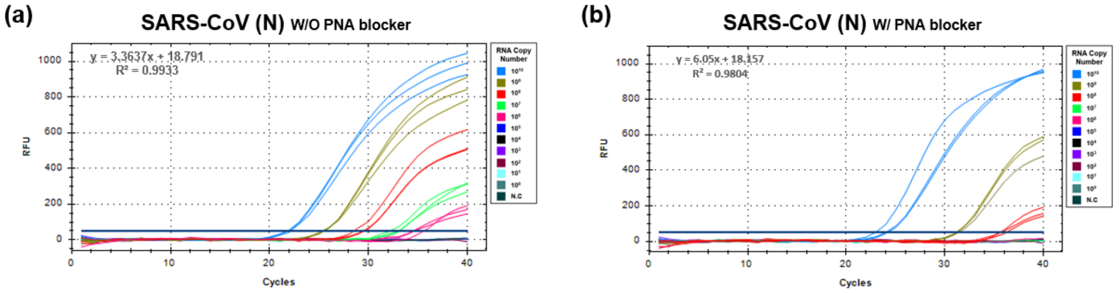

(c)

Mean CT value ± (SD)	RNA copy number											
	1×10^{10}	1×10^{9}	1×10^{8}	1×10^{7}	1×10^{6}	1×10^{5}	1×10^{4}	1×10^{3}	1×10^{2}	1×10^{1}	1×10^{0}	N.C
SARS-COV (N) W/O PNA blocker	21.92±0.18	25.43±0.07	29.18±0.58	32.80±0.54	35.06±0.65	-	-	-	-	-	-	-
SARS-COV (N) W/ PNA blocker	23.71±0.66	31.24±0.11	35.81±0.36	-	-	-	-	-	-	-	-	-

Figure S1. Detection performance of the designed PNA blocker using high SARS-CoV RNA copy numbers. Re-evaluation of the peptide nucleic acid (PNA)-mediated RT-qPCR assay for SARS-CoV N gene amplification with higher RNA copies. Ten-fold serial dilutions (1×10^{0} to 1×10^{10}) of SARS-CoV RNA transcripts without (a) and with (b) PNA blocker were processed for RT-qPCR assay. Each dilution was processed in triplicates and labeled with different colors to identify differences in amplification curves. (c) Positive amplification was determined from the mean cycle threshold values for each RNA dilution point. N.C: negative control; Ct: cycle threshold; SD: standard deviation; '-': not determined.

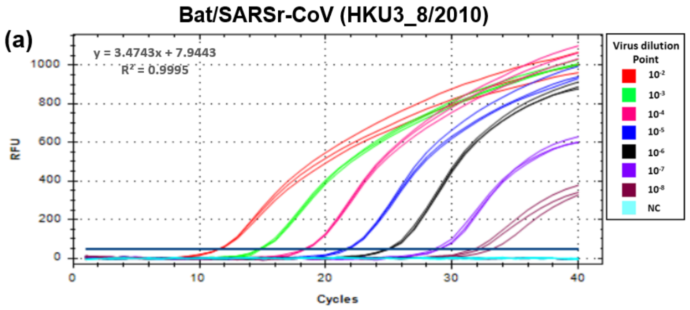

(b)

Mean CT value ± (SD)	RNA copy number								
	1×10^{8}	1×10^{7}	1×10^{6}	1×10^{5}	1×10^{4}	1×10^{3}	1×10^{2}	1×10^{1}	1×10^{0}	N.C
GQ153543/B-CoV/Bat/HKG/HKU3_8/2010 (ORF3ab-E)	11.61±0.05	14.82±0.11	18.39±0.07	21.69±0.17	25.04±0.07	28.92±0.26	32.42±0.73	-	-	-

Figure S2. Detection performance of the ORF3ab-E primer set using a disparate SARSr-CoV strain. Evaluation of the pan-Sarbecovirus primer design for ORF3ab-E gene amplification of Bat/SARSr-CoV (HKU3/2010). Ten-fold serial dilutions (1×10^{0} to 1×10^{8}) of Bat/SARSr-CoV RNA transcripts were processed for RT-qPCR assay. (a) Each dilution was processed in triplicates and labeled with different colors to identify differences in amplification curves. (B) Positive amplification was determined from the mean cycle threshold values for each RNA dilution point. N.C: negative control; Ct: cycle threshold; SD: standard deviation; '-': not determined.

(a)

Mean CT value ± (SD)	Virus dilution point							
	1×10^{-2}	1×10^{-3}	1×10^{-4}	1×10^{-5}	1×10^{-6}	1×10^{-7}	1×10^{-8}	N.C
Virus-1 (N)	17.48±0.02	20.14±0.16	23.17±0.17	26.84±0.18	30.51±0.37	33.91±0.54	-	-
Virus-2 (N)	17.17±0.27	20.04±0.40	23.23±0.51	26.61±0.29	30.37±0.16	35.11±1.18	-	-
Virus-3 (N)	13.94±0.19	17.6±0.23	21.86±1.26	24.74±0.26	28.39±0.14	32.15±0.20	-	-

(b)

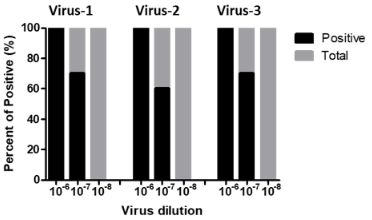

(c)

Virus	Specimen type	Number of s pecimen	Detection percentage (%) NIID-Japan RT-qPCR	Ct value
SARS-CoV-2	Clinical	23	73.9	18.10-36.40

Figure S3. Sensitivity comparison between the dual-target RT-qPCR method and World Health Organization (WHO) recommended RT-qPCR method. Cross-evaluation of intact viruses using WHO-recommended primers. From WHO-recommended primers for SARS-CoV-2 detection, the Japan N-gene primers (32) were used for the cross-evaluation of the RNA extracts from cell-propagated SARS-CoV-2 viruses isolated from patients diagnosed with COVID-19. Intact viral RNA extracts were ten-fold serially diluted (10−2 to 10−8) and processed to detect SARS-CoV-2. (a) Positive amplification was determined from the mean cycle threshold values for each RNA dilution point. (b) Limit of detection was assessed using 10−6, 10−7, and 10−8 dilutions in 10 repetitions. (c) Ct value range detected from the number of positives among the twenty-three SARS-CoV-2 clinical patient samples cross-validated using the NIID-Japan-RT-qPCR detection method. N.C: negative control; Ct: cycle threshold; SD: standard deviation; '-': not determined.
